# Effects of intensivist coverage in a post-anaesthesia care unit on surgical patients' case mix and characteristics of the intensive care unit

**DOI:** 10.1186/cc11428

**Published:** 2012-07-18

**Authors:** Marc Kastrup, Matthes Seeling, Stefan Barthel, Andy Bloch, Marie le Claire, Claudia Spies, Matthias Scheller, Jan Braun

**Affiliations:** 1 Department of Anaesthesiology and Intensive Care, Campus Virchow-Klinikum and Campus Charité Mitte, Charité-University Medicine, Augustenburger Platz 1, 13353 Berlin, Germany; 2 Controlling Department, Charité-University Medicine, Charitéplatz 1, 10117 Berlin, Germany; 3 Hospital Director and Member of the Executive Board, Charité-University Medicine, Charitéplatz 1, 10117 Berlin, Germany

## Abstract

**Introduction:**

There is an increasing demand for intensive care in hospitals, which can lead to capacity limitations in the intensive care unit (ICU). Due to postponement of elective surgery or delayed admission of emergency patients, outcome may be negatively influenced. To optimize the admission process to intensive care, the post-anaesthesia care unit (PACU) was staffed with intensivist coverage around the clock. The aim of this study is to demonstrate the impact of the PACU on the structure of ICU-patients and the contribution to overall hospital profit in terms of changes in the case mix index for all surgical patients.

**Methods:**

The administrative data of all surgical patients (n = 51,040) 20 months prior and 20 months after the introduction of a round-the-clock intensivist staffing of the PACU were evaluated and compared.

**Results:**

The relative number of patients with longer length of stay (LOS) (more than seven days) in the ICU increased after the introduction of the PACU. The average monthly number of treatment days of patients staying less than 24 hours in the ICU decreased by about 50% (138.95 vs. 68.19 treatment days, *P *<0.005). The mean LOS in the PACU was 0.45 (± 0.41) days, compared to 0.27 (± 0.2) days prior to the implementation. The preoperative times in the hospital decreased significantly for all patients. The case mix index (CMI) per hospital day for all surgical patients was significantly higher after the introduction of a PACU: 0.286 (± 0.234) vs. 0.309 (± 0.272) *P *<0.001 CMI/hospital day.

**Conclusions:**

The introduction of a PACU and the staffing with intensive care staff might shorten the hospital LOS for surgical patients. The revenues for the hospital, as determined by the case mix index of the patients per hospital day, increased after the implementation of a PACU and more patients can be treated in the same time, due to a better use of resources.

## Introduction

There is an increasing demand for critical care, which can lead to capacity limitations in the intensive care unit (ICU). These limitations can cause a delay in the admission of patients from emergency departments and the need to postpone elective surgery [1] potentially leading to increased morbidity and mortality [2]. Furthermore, in a fee-for-service system at fixed prices, as is the German Diagnosis Related Group System (G-DRG), prolonged process times and internal cues lead to hospitals having to bear higher costs at equal revenue making them less profitable. On the other hand, DRGs induce the implementation of goal oriented treatment and support discussion of therapeutic aims and quality in patient treatment [3,4]. For hospitals and their staff the challenge is to optimize clinical processes and to optimize the effectiveness of treatment in regard to patient´s outcome.

Operating theatres and the intensive care units are very costly areas of a hospital, so it seems advisable to look at these areas closely [5]. Hospitals are forced to develop new and innovative strategies to address capacity restraints, improve throughput without a large amount of unoccupied beds in the intensive care unit. A decrease in quality of care or premature discharge of the patients from the intensive care unit, which negatively influences mortality [6], must be avoided.

Most major hospitals deliver different levels of intensive care [7]. Several studies have focused on "ICU-appropriateness" issues by retrospective analysis of qualitative ICU-bed utilization. The difficulty is a stratification between low-risk and high-risk patient-days using the Therapeutic Intervention Scoring System (TISS) categories [8] or specifically developed indices of level of care defined by the severity of organ failures [9] or by intensive care interventions [10]. This approach to defining the level of care is helpful in planning which level of care is appropriate for the individual patient, but does not solve the problem of restricted availability of ICU beds for emergency admissions, for example.

When there is no reserve ICU-capacity, intensivists select one of several less suitable options for new patients [11], each having individual risks for the patient. These options include interhospital transfers, initiation of treatment in the emergency department, trial of standard care in a low-acuity ward, cancellation of surgery and early discharge from ICU after triage [12]. Several studies have shown that early or night time discharge from the ICU is associated with higher ICU readmission rates, longer hospital length of stay and increased mortality [13,14]. Some authors describe the transferral to a post anaesthesia care unit (PACU) as an unfavourable option, since equipment, expertise and staffing levels in the PACU are different from the ICU [11].

A possible solution to this problem might be the inclusion of the PACU in the process of distribution of patients to the different levels of intensive care. By staffing the PACU with intensive care staff 24 hours a day, patients can be treated at any time with the same equipment and expertise as in the ICU. The difference is the limitation of the length of treatment to 24 hours and the manpower used in the PACU for treatment, which is less, compared to the ICU. Besides a change of staffing in the PACU, definitions of admission and discharge criteria, which include the maximum duration of stay, were introduced. These admission criteria are used to overcome organizational barriers, as an alternative to routine admission to intensive care after certain types of surgery or trauma. The intention of the intensivist coverage is not an expansion of ICU-capacities, but the provision of high-quality intensive care for all patients without delay or postponement of elective surgery due to a lack of ICU-beds.

The economic aspects for the whole hospital of the introduction of PACU, as an alternative area to treat patients after major surgery, trauma or emergency admissions are not well investigated. There is also some evidence that treatment of post-surgery patients in the PACU is superior to treatment in conventional ICUs. Responsible for this effect might be the early goal-oriented therapy in the PACU and the clinical focus on the fast recovery of postoperative patients [15].

The aim of this study is to evaluate the effect on the structure of ICU-patients and to demonstrate the economic effect of the staffing of a PACU with an around-the-clock intensivist coverage on the hospital revenue for surgical patients of a university hospital.

## Materials and methods

The data protection officer of the hospital approved the collection of the data. The study, analysis and publication were approved by the local ethics committee of the hospital (EA1/314/11), which also waived individual patient consent, due to the retrospective nature of the study and the anonymous structure of the data.

### Organization of care

The PACU is an area within the operating department where patients after any type of surgery or interventions, including cardiac surgery, and patients from the emergency department are treated until the patients can be discharged to a normal ward or an ICU or intermediate care unit (IMCU) when a bed becomes available. There are six beds with complete intensive care monitoring and respiratory care possibilities available. The beds are used for recovery-room patients and PACU-patients.

### Intervention

The PACU is part of the recovery room. During several years there was increasing demand for postoperative monitoring and care of patients. In our institution the staffing of the PACU was changed so that both the nursing and physician staffing are covered by the ICU-team. Outside of the core operating hours of the operating department, the physician staffing was an on-demand presence model. This was changed to a 24-hour in-house critical care physician and nurse presence for the PACU on 1 September 2009. This allows an immediate admission of patients without any delay around the clock. The staffing consists of one physician for all patients and one nurse for a maximum of three PACU patients. This ratio varies during the day as recovery room patients are treated by the same team in the same unit.

The duty of the PACU physician is the allocation of the patients to the PACU, ICU and IMCU beds, in close collaboration with the surgical partners, depending on the clinical condition. If no intensive care bed is available, the patients can be treated in the PACU for up to 24 hours, independent of the degree of organ failure. The department of anaesthesiology and intensive care is in direct charge of 22 ICU beds and treats patients after major surgery or after treatment in the emergency department. Table 1 gives the different admission criteria for the PACU/IMCU and ICU.

**Table 1:** Admission criteria for the PACU, IMCU and ICU

ICU	Planned length of stay >24 hours
	Any form of organ dysfunction, which alone or in combination is a vital threat to the patient
	- cerebral impairment (delirium, intoxications, metabolic disorders, trauma, stroke)
	- respiratory insufficiency with or without hypoxia
	- cardiac failure including vital rhythms disorders
	- shock and/or severe sepsis
	- massive blood loss
	- acute renal failure
	- continuous artificial organ support
PACU	Planned length of stay <24 hours
	- Same indications as for ICU and IMCU, with an expected length of stay under 24 hours
	- Postoperative ventilation or monitoring for >120 minutes

IMCU	Non-ICU, Non-PACU
	- patients with increased monitoring demands or intensive nursing demands
	- therapy of stabile organ dysfunction
	- example: low dose vasopressor therapy, intermittent dialysis treatment, intermittent CPAP-therapy, (no invasive ventilation in IMCU)

Postoperative/ Recovery-room patients	Patients with mild organ dysfunction, which are expected to be discharged to a normal ward within two hours

### Patients

Data from all patients undergoing a surgical procedure at the Charité - University Hospital Campus Mitte between 1 January 2008 and 30 April 2011 were evaluated. Since numerous children are treated in the PACU, for example, after orthopaedic surgery, they were included in the analysis. Ambulatory surgical patients were not included in this evaluation.

### Data collection

All clinically relevant data, including all scores, vital parameters, lab values and medications, are documented in a patient data management system (PDMS) system and can be extracted for evaluations. Every patient admitted to the ICU is included in the system (COPRA-System^® ^GmbH, Sasbachwalden, Germany) 24 hours after discharge of a patient the electronic patient record is changed into a read-only mode and cannot be modified in any way.

Besides clinical data, data from the hospital administrative system are used. The central controlling department of the university collects all relevant data for administrative reasons. All DRG-relevant data are extracted from the administrative data system (i.s.h.med^® ^, Siemens AG, München, Germany). All patients with an operative procedure were included in this study. Patients were excluded if they experienced hospital readmission for the same reason as the initial admission. These patients are summarized in one administrative case and the preoperative time in the hospital would not be correctly calculated.

The readmission rate for ICU readmissions was collected from the hospital administrative system. All patients discharged from an ICU to a normal ward, which were readmitted within 48 hours to an ICU, IMCU or PACU are considered as readmissions.

For the reimbursement of the German hospitals, a performance-orientated and global compensation system is used. The basic principle of this system is the G-DRG-System (German-Diagnosis Related Groups-System), where every hospital case is paid for by a lump compensation. The Institute for the Hospital Remuneration System (InEK GmbH, Siegburg, Germany) performs the administrative tasks during the introduction and continuous development of this system. This institute also proposed a matrix for the distribution of the reimbursement within different areas and subspecialties of a hospital. As there is no widespread cost-centre accounting in German hospitals, this matrix is used for the allocation of revenues within the hospital departments and was also used in this study.

Descriptive statistics comparing the structures and processes of care are expressed as a percentage, mean or median. Appropriate statistical tests, depending on the distribution of the variables, were used to compare the groups. Unpaired Student *t-*test, Pearson chi^2 ^test for independence and Mann-Whitney-U test were used. For the calculations SPSS Version 19 (IBM, Armonk, NY 10504, USA) was used.

## Results

The data of 51,090 patients, who underwent surgery as in-patients in the time period between 1 January 2008 and 30 April 2011 were evaluated. Of these patients, 3,317 were treated in the PACU for more than two hours. During this time period, 5,969 patients were treated in the ICU of the department of anaesthesiology and operative intensive care.

### Impact on ICU characteristics

**Figure 1:**
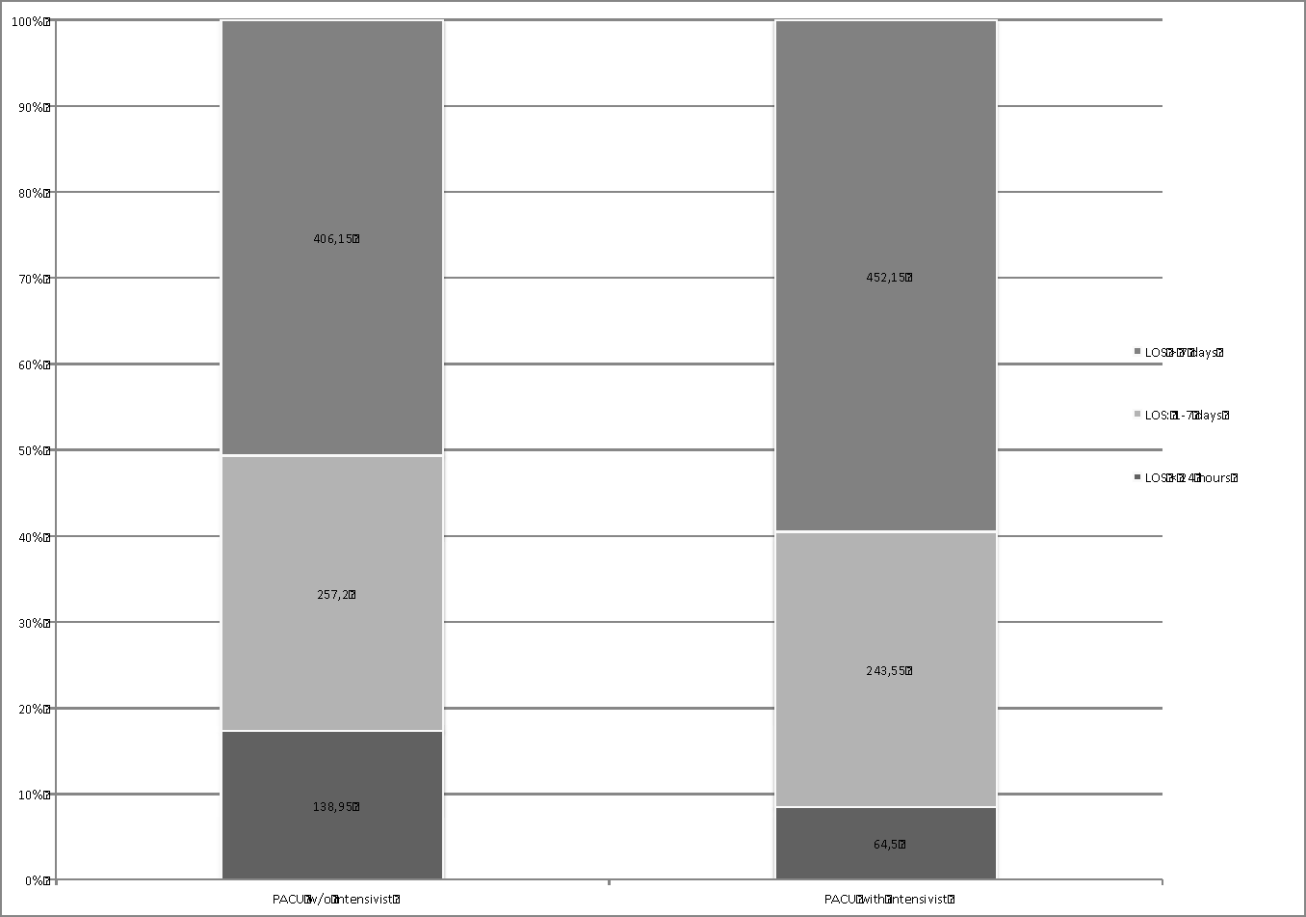
**Distribution of patients by LOS in the ICU before and after the introduction of the PACU**. Values are mean number of treatment days per month. *P*-values: LOS <24 hours <0.001, LOS 1 to 7 days: *P *= 0.31, LOS >7 days: *P *= 0.001, PACU w/o intensivist (20 months): n = 3,293 patients, PACU with intensivist (20 months): 2,676 patients.

The impact of the introduction of a PACU-service on ICU-patients' characteristics is shown in Table 2. The mean number of cases treated per month in the ICU dropped significantly from 164.65 (± 14.37) to 133.80 (± 19.42) (*P*-value: <0.001) cases/month, whereas the mean number of treatment days per month did not change significantly. A possible reason for this can be seen in Figure 1, where the distribution of patients according to the length of stay in the ICU is shown. The relative number of patients with longer LOS (more than seven days) increased after the introduction of the PACU, whereas the average number of treatment days of patients staying less than 24 hours in the ICU decreased by about 50% (138.95 vs. 64.5) mean days per month, *P *<0.005).

**Table 2:** General descriptive variables for the ICU, before and after the introduction of the PACU

Group	PACU w/o intensivist		PACU with intensivist		*P*-value
	**Mean per month**	**Std. deviation**	**Mean per month**	**Std. deviation**	
**Cases treated **	164.7	14	133.8	19	<0.001
**Treatment days **	775.9	25	760.2	31	0.091
**Readmission <48 h (%)**	2.55	1.4	1.7	0.9	0.063
**Non-Survivors (deaths)**	8.7	2.9	7.9	3.2	0.369
**SAPS sum**	28,012.5	2,086	27,331.0	3,293	0.758
**Sum points DRG-code 8.980**	23,026.2	8,501	33,098.0	2,295	<0.001
**ARDS days with Prostaglandin-application**	4.5	6.2	9.2	6.8	0.003
**ARDS days with NO-Application **	10.5	13.8	42.7	35.7	<0.001
**Days with extracorporeal gas-exchange**	57.2	124.7	221.6	182.1	0.001
**Ventilation hours (h for all patients, monthly sum)**	10,132.0	1,108	11,243.3	876	0.002
**Dialysis hours (h for all patients, monthly sum)**	230.9	51.1	253.6	56.0	0.204
**Lethality (non-survivors in % from all treated cases)**	5.4	2.0	5.9	2.4	0.512

Pre-PACU (20 months): n = 3,293 patients, post-PACU (20 months): 2,676 patients, the values are the mean values for a month in the given time period.

ARDS, acute respiratory distress syndrome; NO, nitric oxide; PACU, post anaesthesia care unit; SAPS sum, sum of simplified acute physiology score points.

**Figure 2:**
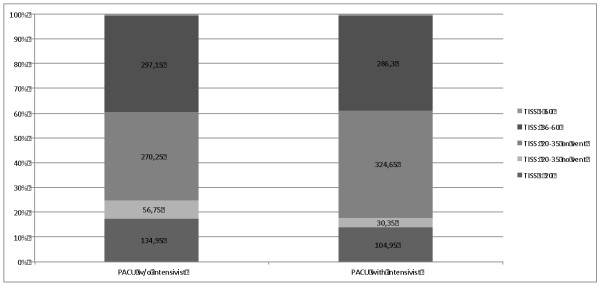
**Distribution of the patients of a month according to the TISS-28 score**. Average number of patient days per month before and after the introduction of a PACU staffing with an intensivist. *P*-values: TISS <20: <0.001; TISS 20 to 35 no vent: <0.001; TISS 20 to 35 on vent: <0.001; TISS 36 to 60: n.s.; TISS >60: n.s.

The patients treated in the ICU seem to be sicker after the implementation of the PACU. Figure 2 gives the average distribution of the patients of a month, according the TISS-28 score, before and after the instruction of the PACU with an intensivist. Since 2007, the German DRG system allows the coding of intensive care as DRG-Procedure, making the severity of disease relevant for reimbursement [16]. The procedure "complex intensive care treatment" is based on several scores, which are collected within the PDMS system. There is a significant increase in the sum of the scores of all the patients, from a monthly mean value of 23.026 to 33.097 (*P *= 0.001) score-points. There is an increase of patient days, where nitric oxide for severe pulmonary dysfunction or right-heart insufficiency was used. The average monthly days with extra-corporal gas exchange devices and renal replacement therapy increased. Despite the increase of severity of disease, the mortality rate of the ICU did not change. The change in the readmission rate did not reach statistical significance after the introduction of the PACU (Table 2).

**Figure 3:**
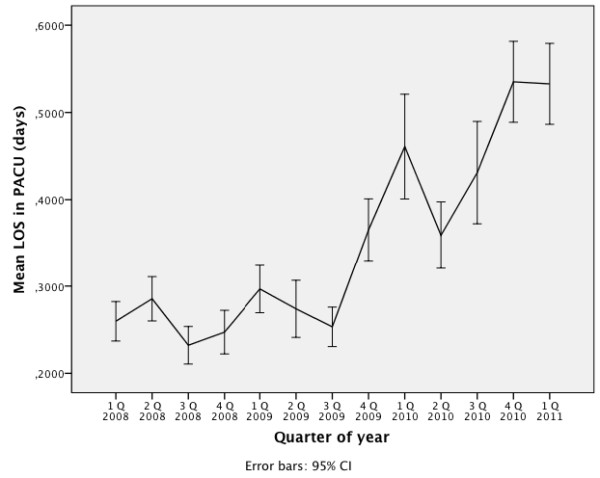
**Mean length of stay in PACU for all PACU-patients in days**. Data for quarters of year, from 1 January 2008 to 30 April 2011; n = 3,317 patients.

Figure 3 gives the mean length of stay for all patients treated in the PACU for each yearly quarter from 2008 to the first quarter of 2011. After the introduction of intensive care staffing there is an increase in the length of stay for the patients treated in this area.

### Effects on hospital efficiency

**Figure 4:**
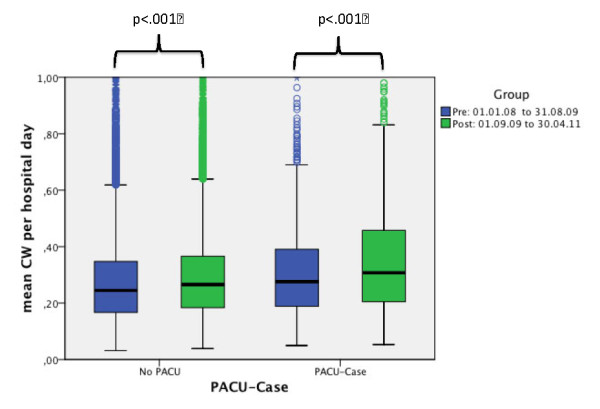
**Mean CW per hospital day, before and after staffing the PACU with an intensivist**. Values for all surgical patients treated in the PACU and surgical patients without treatment in PACU. PACU-Cases n = 3,317, no-PACU cases: n = 47,773.

The impact on various hospital efficiency data is shown in Table 3. More patients were treated in the PACU for a longer period of time. The mean LOS in the PACU after the full implementation of the PACU was 0.45 (± 0.41) days, compared to 0.27 (± 0.2) days prior to the implementation. The preoperative time, defined as the time period from the admission to the hospital and the onset of the surgical procedure, decreased significantly for all patient-groups, independent of the fact if they were treated in the ICU, the IMCU, the PACU or were just regular surgical cases. The overall length of hospital stay decreased significantly for all surgical patients from 8.3 (± 11.8) to 7.71 (± 10.99) days. To determine the efficiency of the hospital, the case mix index (CMI) per hospital day was calculated. The CMI was significantly higher in the period after the introduction of a PACU: from 0.286 (± 0.234) to 0.309 (± 0.272) *P *<0.001 CMI/hospital day. In Table 3, the distribution of the case mix index between the ICU, normal wards and other parts of the hospital according to the matrix provided by the InEK-institute for each corresponding DRG of the patients is given. Comparing the time period before the PACU with the time after the PACU, the average case mix index in the normal ward decreased and the average case mix index for the ICU per day increased. Figure 4 shows the CMI per hospital day for PACU and non-PACU patients for the time periods before and after the introduction of a PACU.

**Table 3:** Data from the hospital information system

Group	PACU w/o intensivist (n = 24,972)			PACU with intensivist (n = 26,118)			
	**n**	**mean**	**Std. Deviation**	**n**	**mean**	**Std. Deviation**	**Asymptotic significance (2-tailed)**

LOS in PACU (days)	1,554	0.27	0.20	1,763	0.45	0.41	<0.001

LOS in ICU (all types of ICU´s)(days)	3,877	6.04	13.36	3,778	7.12	16.37	<0.001

Pre operative days (all patients)		1.69	4.02		1.56	3.75	<0.001

Pre operative day (PACU-patients)		2.53	4.89		2.34	4.62	<0.001

Pre operative day (ICU-patients)		3.78	6.88		3.56	6.13	0.002

days on normal ward		7.34	9.25		6.65	7.82	<0.001

LOS hospital (days)		8.30	11.80		7.71	10.99	<0.001

CMI (InEK) normal ward		0.659	0.512		0.644	0.492	0.042

CM (InEK) ICU		0.399	2.512		0.424	2.844	<0.001

CM (InEK) other parts of InEK Cost-matrix		0.957	1.269		1.023	1.320	<0.001

CW per hospital day (overall)		0.286	0.234		0.309	0.272	<0.001

PACU w/o intensivist: 1 January 2008 to August 31 2009, PACU with intensivist: 1 September 2009 to 30 April 2011 (20 months prior to PACU and 20 months post PACU).

CMI, case mix index; CW, cost weight; ICU, intensive care unit; InEk, Institute for the Hospital Remuneration System; LOS, length of stay; PACU, post anaesthesia care unit

## Discussion

The results of our study show that the implementation of PACU with a 24-hour intensive care physician can influence the patient structure of the intensive care unit and can be beneficial for the hospital in terms of better management of resources. The fraction of long-term patients in the ICU increased. The patients in need of short-term intensive care up to 24 hours were treated in the PACU, creating resources in the intensive care unit. The more efficient short-term treatment in the PACU and the efficient allocation of capacity for long-term ICU-treatment in the ICU may result in an overall increase of the CM per bed per day for all surgical patients.

Several studies have focussed on the concept of "fast-track" treatment of patients after major abdominal or cardiac surgery [15,17-19], which includes an optimal preoperative assessment and preparation of the patient, optimal choice of anaesthetics, techniques and prophylactic drugs to minimize the impact of surgery and to maintain and recover optimal organ function after surgery. The objective of these concepts is the reduction of length of stay in the ICU, and improvement of outcomes for patients. Most of these protocols, especially for cardiac surgery patients, have been implemented in the ICU. Ender and co-workers [15] were able to demonstrate that patients after cardiac surgery fast-track treatment can be effectively managed in a post-anaesthetic care unit with improved outcomes. Most fast-track concepts include anaesthetic techniques, which are aimed at quick recovery of the patient and optimal pain therapy. In contrast to these studies, in our study all surgical patients were analysed. In the allocation of patients postoperatively, the type of anaesthesia only plays a minor role, as the PACU can provide the same level of care, as an ICU can supply. In other studies, "fast-track"-patients are identified prior to surgery by well-defined criteria and receive numerous interventions aimed at a fast recovery of the patient.

Studies report that about 12.4 to 14% of all elective surgeries are postponed [20,21]. One major problem for many hospitals cannot be improved by the implementation of "fast-track" surgery: the deficiency of intensive care beds. This lack of capacities leads to a postponement of elective surgery or undesirable waiting times for optimal treatment for patients admitted to the emergency department after major trauma. The problem of reduced access to intensive care can be addressed with certain interventions, but these might also be responsible for increased mortality rates and length of stay [12]. The unavailability of a postoperative intensive care bed is not the only reason for the cancellation of surgery. In our institution, after the introduction of the PACU, no surgical case was cancelled due to a lack of postoperative care for the patient. The mean time from admission to the hospital to initiation of surgery dropped for all patients, independent of whether they were treated in the PACU or ICU or neither or both.

Other hospitals have introduced acuity adaptable patient care units to fast track postoperative patients in an effort to reduce hospital length of stay [22]. These novel models do not address the problem of limited ICU-bed resources though, and have little potential to reduce the number of delayed surgeries due to a lack of ICU capacities.

A worldwide study by Rothen and colleagues [23] conducted in 275 intensive care units demonstrated a considerable variability in outcomes and resource use. The secondary aim of this study was to assess whether outcome or resource use are related to ICU structure and process. In the multivariate analysis in the Rothen study, only interprofessional rounds, emergency department and geographical location were significant. The results from this study on 16,560 adults imply that some other confounding factors and not factors, such as staffing, specialists per bed or type of hospital, play an important role. In a benchmark study by Zimmerman and colleagues [24] using the data of over 350,000 patients from 108 ICUs, the hospitals with the shortest ICU and hospital stay had alternatives to intensive care, methods to facilitate patient throughput and used multiple protocols for high-volume diagnoses. The results of these studies demonstrate that only a number of interventions can be effective, when it comes to influencing the resource use and outcomes of an ICU.

A study by Rapoport and colleagues [25] investigated the length of stay data as a guide to hospital economic performance for ICU-patients and demonstrated that the first day of ICU-treatment is about four to five times more expensive than post-ICU days and more expensive than the following ICU days. The results from our study demonstrate that the patients in the ICU have longer lengths of stay and seem to be sicker and need more artificial invasive organ support (extra corporal cardiac assist devices, renal replacement therapy). This suggests a more effective use of the expensive resource ICU. The short-stay patients with stays under 24 hours are increasingly treated in the PACU and the overall length of hospital stay for all surgical patients was significantly shorter in the time period after the introduction of the PACU. At the same time, the outcome variable ICU-readmission improved. The mortality for all surgical patients did not change (data not shown).

Whether the intensive treatment in the PACU with the goal to meet the discharge criteria within 24 hours has directly influenced the overall length of hospital stay is speculative. As planned, PACU patients should meet discharge criteria at the end of the treatment time, the staff of the PACU uses various aspects of fast-track surgery to meet these goals: rapid achievement of normothermia, rapid extubation if the patient meets extubation criteria, intensive use of CPAP-therapy if indicated, fluid management to achieve fast hemodynamic stability and early oral intake of postoperative patients. Of course, patients are not discharged and are transferred to an ICU or IMCU if they do not meet the discharge criteria within this time. Ender and colleagues [15] implemented a cardiac surgery fast track treatment in the PACU and demonstrated pronounced results for shorter duration of ventilation, time in PACU or ICU and hospital length of stay, with at the same time improved outcome parameters, like mortality. This study has some differences compared to our situation: the PACU was used during daytime only for the fast-track patients and staffed by the anaesthesia department. Our PACU runs 24 hours a day and all patients in need of intensive care are treated there. The patients treated in our PACU are not selected towards specific types of anaesthetic management.

We did not directly compare our lengths of stay with other data from the literature but only within the time development, as the different health care systems make comparisons difficult. The German-DRG-system defines minimum length of stay in the hospital and hospitals receive less reimbursement if patients are discharged before these defined times, making postoperative length of stay difficult to compare. Additionally, the intensive care team has little influence on the discharge decision on the general ward.

One effect that was not investigated in our study was the impact of the PACU on vacant intensive care beds, as this is not an issue of debate in our department. With the introduction of the PACU, there is no need for the reservation of a bed within the ICU for unexpected emergency cases, as there are always capacities for unplanned or emergency department patients.

Our study differs from many other studies on the effectiveness of intensive care therapy. We used a global view from the general hospital perspective. We included all surgical patients in our evaluation, excluding only patients (n = 1,448) readmitted for the same reason after discharge from the hospital as the administrative data of these patients are combined in one case. As other authors already have noted [23], the variability in outcome and resource use is a very complex structure, which is influenced by numerous factors. We included all surgical patients, as an optimal treatment process for some patients might influence other patients, which are not directly affected. If the hospital length of stay is reduced for some patients, the hospital can use these resources for treatment of new cases and increase the volume of treated patients and perhaps influence the profit. As the base rate has changed over the study period, we used the value of the case-mix to assess the financial impact of structural changes, as the case mix index is used for determination of the revenue for the hospital. Our results demonstrate that despite the fact that surgical patients in need of intensive care stayed longer in the ICU, the overall effect was a reduction of LOS for surgical patients and an increase of case mix index per hospital day. For the subgroup of all ICU-patients, a shorter duration of hospital stay could also be demonstrated (data not shown). This enables the surgical partners to treat more patients in the same number of beds over a given time with more revenues for each hospital day.

### Limitations

Our study has several limitations. First of all, we used administrative data. Due to rigorous routine data validation procedures the quality of data can be assumed as high. However, missing data cannot be excluded and not quantified, especially concerning the lengths of stay in the PACU, when patients are not registered in the hospital administrative system. Second, the implementation of the PACU was a gradual process; in the beginning the PACU was only restricted to postoperative patients (for a maximum of two hours) and the anaesthesia nurses cared for the patients. In a second step, patients were allowed to stay longer in the PACU, and a physician was in charge of these patients. The next step was the complete implementation around the clock, and nurse and physician staffing with ICU staff. Third, patients are not treated uniquely in the PACU; the waiting time after surgery or emergency admission is covered in the PACU until an ICU bed becomes available. Other patients do not meet the discharge criteria and must be transferred to an ICU. This makes it difficult to attribute certain effects to certain parts of the care process. Fourth, due to the absence of cost-centre accounting, there is no direct information about the individual patient. Other factors than the PACU certainly also influenced the length of hospital stay, but these cannot be quantified. There are no data available for long-term effects, such as 30-day mortality, as German data security laws prohibit the acquisition of patient data by, for example, the health insurance companies for studies like ours.

## Conclusions

The introduction of a PACU and the staffing with intensive care staff might shorten the hospital length-of-stay for surgical patients. The revenues for the hospital, as determined by the case mix index of the patients per hospital day, increased after the implementation of a PACU, including the staffing with an around the clock intensive care specialist. More patients can be treated in the same time, due to a better use of resources.

## Key messages

• The staffing of a PACU with an intensivist allows admission of emergency and unexpected patients to units with the possibility of intensive care without any delay.

• The preoperative time on the ward can be reduced by a PACU, as no surgical cases are postponed due to a lack of postoperative intensive care treatment.

• The CMI per hospital day for all surgical patients and for patients treated in the PACU increased.

• The resources in the ICU are used by long-stay ICU-patients with more organ failures and more need for invasive therapies.

• The short-term ICU patients with stays of less than 24 hours are treated in the PACU without an increased readmission rate.

## Abbreviations

CMI: case mix index; DRG: diagnosis related groups; G-DRG-System: German-Diagnosis Related Groups-System; ICU: intensive care unit; IMCU: intermediate care unit; LOS: length of stay; PACU: post anaesthesia care unit; PDMS: patient data management system; TISS: Therapeutic Intervention Scoring System.

## Competing interests

The authors declare that they have no competing interests related to this article. Prof. Claudia Spies got funding from Merck Sharp und Dome GmbH, Astra Zeneca, Bristol-Myers Squibb GmbH, Pfizer and Fresenius Kabi.

## Authors' contributions

MK developed the study design, performed the interpretation of the data and drafted the manuscript. MSeeling and MScheller revised the manuscript critically for important intellectual content. SB, AB and MlC performed the data acquisition, helped in the study design and have been involved in the drafting of the manuscript. JP and CS were involved in the study design, the interpretation of the results and revised the manuscript critically for important intellectual content. All authors have given final approval of the version to be published.
